# Prediction of pKa Values for Neutral and Basic Drugs based on Hybrid Artificial Intelligence Methods

**DOI:** 10.1038/s41598-018-22332-7

**Published:** 2018-03-05

**Authors:** Mengshan Li, Huaijing Zhang, Bingsheng Chen, Yan Wu, Lixin Guan

**Affiliations:** College of Physics and Electronic Information, Gannan Normal University, Ganzhou, Jiangxi 341000 China

## Abstract

The pKa value of drugs is an important parameter in drug design and pharmacology. In this paper, an improved particle swarm optimization (PSO) algorithm was proposed based on the population entropy diversity. In the improved algorithm, when the population entropy was higher than the set maximum threshold, the convergence strategy was adopted; when the population entropy was lower than the set minimum threshold the divergence strategy was adopted; when the population entropy was between the maximum and minimum threshold, the self-adaptive adjustment strategy was maintained. The improved PSO algorithm was applied in the training of radial basis function artificial neural network (RBF ANN) model and the selection of molecular descriptors. A quantitative structure-activity relationship model based on RBF ANN trained by the improved PSO algorithm was proposed to predict the pKa values of 74 kinds of neutral and basic drugs and then validated by another database containing 20 molecules. The validation results showed that the model had a good prediction performance. The absolute average relative error, root mean square error, and squared correlation coefficient were 0.3105, 0.0411, and 0.9685, respectively. The model can be used as a reference for exploring other quantitative structure-activity relationships.

## Introduction

As an important step in drug design, the quantitative structure-activity relationship (QSAR) study has become one of the most active branches because it can improve the efficiency of drugs by computer simulation and provide ideas for designing new drugs^1^. The QSAR study is also important in computer science, chemistry, pharmacy and life sciences^2^. The efficacy of drugs is mainly achieved by activating the acidity coefficient, which is called pKa constant and denotes the capability of an acid to dissociate hydrogen ions^3,4^. The pKa value is an important parameter in drug design and determines pharmacological activity. The experimental measurement method of pKa value is relatively cumbersome and time-consuming. Therefore, it is necessary to establish an accurate and efficient pKa prediction model^5,6^.

Model establishment is one of the key steps in QSAR research. The traditional model establishment methods include linear regression and least square method^7,8^. The modern computing methods consist of support vector machines (SVM)^9^, artificial neural networks (ANN)^10–13^, and various intelligent algorithms^14,15^. Polanski^16^ proposed a model utilizing ANN and partial least squares (PLS) to study the relationship between molecular surface area and pKa value and predicted the pKa values of aromatic acids and alkyl acids. Luan^17^ proposed a pKa prediction model based on the heuristic method (HM) and radial basis function artificial neural network (RBF ANN) and obtained the better prediction performance. Previous studies confirmed that the ANN model had the better performance in QSAR modelling^18,19^, but the performance of ANN depended on its training algorithm. The training algorithm plays a decisive role in RBF ANN model and various evolutionary algorithms have been successfully applied in the training of RBF ANN^20–23^.

The selection of molecular descriptors largely determines the quality of QSAR model^24–26^. There are many selection methods of molecular descriptors, which can be mainly divided into two categories: traditional stepwise selection methods (including the PLS method and its variants) and the modern search algorithm based on optimization strategy^27,28^. The first category is simple, direct, and efficient, but it fails to achieve the global optimum, especially in the complex data sets. The second category is a global optimal method and shows significant advantages. It is easy to search the optimal solution and suitable to deal with complex large data sets. Therefore, the second category has become one of the hotspots^29^.

Several commonly used evolutionary algorithms, such as genetic algorithm, particle swarm optimization algorithm, ant colony algorithm, and firefly algorithm, have been successfully applied in the modeling of QSAR and the selection of molecular descriptors^30–32^. However, evolutionary algorithms have many shortcomings, such as premature convergence, and slow local search and the developed QSAR models are unsatisfactory. Therefore, an improved PSO algorithm, called CSAPSO-EDCD algorithm, based on population entropy diversity, convergence/divergence strategy and the self-adaptive adjustment strategy of weight factor was proposed. The CSAPSO-EDCD algorithm was applied in RBF ANN training and the selection of molecular descriptors in order to develop an efficient and accurate hybrid intelligent QSAR model for predicting the pKa values of neutral and basic drugs and exploring other QSAR models based on protonation changes upon the binding^33–39^ and molecular fingerprint similarity search^40–43^.

## Theories and Methods

The model proposed in this paper involves several theories: RBF ANN, PSO and its improved algorithm.

### RBF ANN

Radial basis function artificial neural network (RBF ANN) is one of the most widely used forward neural network models^44,45^. It has three layers of network structures: input layer, hidden layer, and output layer. The activation function adopted in the paper is the gauss function and defined as:

1 $${{g}}_{{i}}({{x}}_{{k}})=\exp (-\frac{{\Vert {{x}}_{{k}}-{{c}}_{{i}}\Vert }^{2}}{{\sigma }_{{i}}^{2}}),$$

where ${x}_{k}(1\le k\le n)$ is the *k*th output vector; ${c}_{i}(1\le i\le c)$ is the basis function center; *σ*_*i*_ is the spreading constant; *n* is the number of samples; *c* is the number of hidden nodes. The network output is defined as:

2 $$O({x}_{k})=\sum _{i=1}^{c}{w}_{i}\,{g}_{i}({x}_{k}),$$

where *w*_*i*_ is the connection weight of the *i*th hidden node.

However, the artificial neural network has many problems to be solved. For example, the performance is directly related to the optimization of the network weight. The training process of RBF ANN can be considered as the optimization process of function center, spreading constant and connection weight, namely, *c*_*i*_, *σ*_*i*_, *w*_*i*_.

### Improved PSO Algorithm

#### Standard PSO algorithm

PSO is a widely applied population evolutionary algorithm proposed by Eberhart and Kennedy^46,47^ and characterized by fast convergence, simple parameter adjustment and easy realization. The standard PSO algorithm updates its own speed and position, as expressed in Eqs (1) and (2):

3 $${{\rm{v}}}_{{\rm{i}},{\rm{d}}}^{{\rm{k}}+1}={\omega }{{\rm{v}}}_{{\rm{i}},{\rm{d}}}^{{\rm{k}}}+{{\rm{c}}}_{1}({{\rm{p}}}_{{\rm{i}},{\rm{d}}}^{{\rm{k}}}-{{\rm{x}}}_{{\rm{i}},{\rm{d}}}^{{\rm{k}}})+{{\rm{c}}}_{2}({{\rm{p}}}_{{\rm{g}},{\rm{d}}}^{{\rm{k}}}-{{\rm{x}}}_{{\rm{i}},{\rm{d}}}^{{\rm{k}}});$$

4 $${{\rm{x}}}_{{\rm{i}},{\rm{d}}}^{{\rm{k}}+1}={{\rm{x}}}_{{\rm{i}},{\rm{d}}}^{{\rm{k}}}+{{\rm{v}}}_{{\rm{i}},{\rm{d}}}^{{\rm{k}}+1};$$

where *i* = 1, …, m; *ω* is the inertia weight factor, which controls the inertia of the particles and possesses the capability of expanding search space; C_1_ and C_2_, which are the learning factors, represent the statistical acceleration weight when each particle arrives at the extreme-value position; ${{\rm{v}}}_{{\rm{i}},{\rm{d}}}^{{\rm{k}}}$ and ${{\rm{x}}}_{{\rm{i}},{\rm{d}}}^{{\rm{k}}}$ respectively denote the velocity and position of the *i*-th particle in the *d*-dimensional space at *k-*th iteration; ${{\rm{p}}}_{{\rm{i}},{\rm{d}}}^{{\rm{k}}}$ is the local best position of *i-*th particle in the *d*-dimensional space; ${{\rm{p}}}_{{\rm{g}},{\rm{d}}}^{{\rm{k}}}$ is the global best position of the population upon arriving at the *d*-dimensional space.

However, PSO algorithm cannot ensure the optimal solution in each execution. In order to obtain the optimization network parameters, an improved PSO algorithm, called CSAPSO-EDCD, based on population entropy diversity, convergence/divergence strategy and the self-adaptive strategy, has been developed in this paper and then applied in the optimization of the function center, spreading constant and connection weight of RBF ANN.

#### Chaotic self-adaptive strategy

Thus, in this study, the chaotic self-adaptive PSO algorithm, or CSAPSO algorithm, is deduced by applying Lorenz chaos equations and self-adaptive strategies in the adjustment of the learning factors and inertia weight in the PSO algorithm. The inertia weight factor, *ω*, is changed to Eq. (5).

5 $$\omega ={\omega }_{\max }-\mathrm{Pgbest}(k)/{{\rm{Plbest}}}_{{\rm{ave}}}-({\omega }_{\max }-{\omega }_{\min })\times k/{k}_{\max },$$

where ω_max_ and ω_min_ respectively denote the maximum and minimum inertia weights; Pgbest(k) denotes the global best fitness in the *k-*th iteration; Plbest_ave_ denotes the average local best fitness; k_max_ denotes the maximum number of iterations; and k denotes the current iteration.

The learning factors *C*_1_ and *C*_2_ are obtained with the chaotic sequences generated by the Lorenz equations in Eq. (6).

6 $$\{\begin{array}{c}\frac{dx}{dt}=-a(x-y)\\ \frac{dy}{dt}=rx-y-xz\\ \frac{dz}{dt}=xy-bz\end{array},$$

where *a*, *b*, and *r* are the positive control parameters. When *a*, *b*, and *r* are respectively set to be 10, 8/3, and 28, the learning factors (i.e, *c*_1_ and *c*_2_) are in a chaotic state and defined as follows:

7 $$\{\begin{array}{c}{c}_{1}=x(t)\\ {c}_{2}=y(t)\end{array}.$$

Chaotic variables are characterized by randomness, ergodicity, and regularity. By changing the characteristics of chaotic variables, the algorithm can simultaneously increase population diversity and solve the premature convergence problem.

#### Population entropy diversity strategy

Entropy is used to describe the state of a system and indicate the uncertainty in the system or the degree of confusion. The level of entropy can directly reflect the degree of chaos of a system. The higher the entropy is, the more chaotic the system is. On the basis of the definition of entropy in information theory, the population entropy is used to describe the population diversity in the PSO algorithm.

##### Definition

Population entropy. Population size is set to be N. *Q* disjoint subgroups $\{{s}_{1}^{t},{s}_{2}^{t},\mathrm{...},{s}_{Q}^{t}\}$ exist in the *t*-th iteration. The number of particles in each subgroup is described as $\{|{{\rm{s}}}_{1}^{{\rm{t}}}|,|{{\rm{s}}}_{2}^{{\rm{t}}}|,\mathrm{...},|{{\rm{s}}}_{{\rm{Q}}}^{{\rm{t}}}|\}$. Thus, the population entropy in the *t*-th iteration is defined as follows:

8 $${E}_{t}=-k\sum _{i=1}^{Q}{p}_{i}^{t}{\mathrm{log}}_{2}({p}_{i}^{t}),\,{\rm{where}}\,{p}_{i}^{t}=\frac{|{s}_{i}^{t}|}{N}.$$

The population entropy reflects the distribution situation of the population particles. When the population entropy is higher, the particles are in a more chaotic state. The more uniformly distributed the particles are in space, the better the population diversity is. Conversely, the lower the population entropy is, the less chaotic the population is. In this situation, the population particles may converge in the nearest region with one or few extreme points and the population diversity is poor.

In order to evaluate population diversity with population entropy, maximum threshold and minimum threshold are respectively set as *E*_*high*_ and *E*_*low*_. If the population entropy is between *E*_*low*_ and *E*_*high*_, then the population is in the equilibrium state. If the population entropy is higher than *E*_*high*_, the population is in a state of exploration. If the population entropy is lower than *E*_*low*_, the population is in a local search state.

#### Convergence/divergence strategy

In the convergence and divergence strategies, the necessary conditions for the convergence and divergence of the particles should be determined. In a previous study, the conditions of convergence depend on the inertia weight and learning factor^48,49^. When the inertia weight and the sum of two learning factors are less than 1 and 3, respectively, the particles are always convergent. For example, when the inertia weight and the sum of the two learning factors are respectively 0.65 and 0.1, the particles rapidly converge. When the inertia weight is greater than 1, the particles are always divergent. The higher the inertia weight is, the faster the spread rate of the particles is. In the study, Eqs (9) and (10) respectively express the control strategies of convergence and divergence.

9 $$\{\begin{array}{c}\omega =0.65|\sin (\frac{1}{{E}_{t}})|\\ \phi =0.1|\sin (\frac{1}{{E}_{t}})|\end{array};$$

10 $$\omega =1+\lambda \phi \frac{1}{{E}_{t}};$$

where *ω* indicates the inertia weight; *E*_*t*_ indicates the population entropy; *φ* is the sum of *c*_1_ and *c*_2_; *λ* is the positive divergence coefficient and set to be 2.

#### CSAPSO-EDCD algorithm

The CSAPSO-EDCD algorithm is deduced by combining the convergence/divergence and chaotic self-adaptive strategies on the basis of the diversity of the population entropy. When the population entropy is higher than the set maximum threshold, *E*_*high*_, and the particles are in the exploration state, the CSAPSO-EDCD algorithm uses the convergence strategy to induce the particle to move to group center. When the population entropy is lower than the set minimum threshold, *E*_*low*_, and the particles enter the state of exploitation, the algorithm uses the divergence strategy to force the particle to move away from group center. If the population entropy is between *E*_*low*_ and *E*_*high*_, the existing search strategy is maintained. The procedure of CSAPSO-EDCD algorithm is shown as follows:

Step 1: Initialization. Initialize the population size, the maximum and minimum numbers of iterations, the maximum and minimum population entropy, etc.

Step 2: Fitness evaluation. Calculate the fitness value of each individual.

Step 3: Update the extreme value.

Step 4: Population entropy evaluation.

if (the population entropy is greater than the max population entropy *E*_*high*_) then

Particles with the convergence strategy

else if (the population entropy is less than the min population entropy *E*_*low*_) then

Particles with the divergent strategy

else

Particles with the chaotic self-adaptive strategy

end if

Step 5: Finished. Confirm whether the iterative conditions are satisfied. If they are satisfied, then the evolution is finished, otherwise jump to Step 2 to continue.

**Figure 1:**
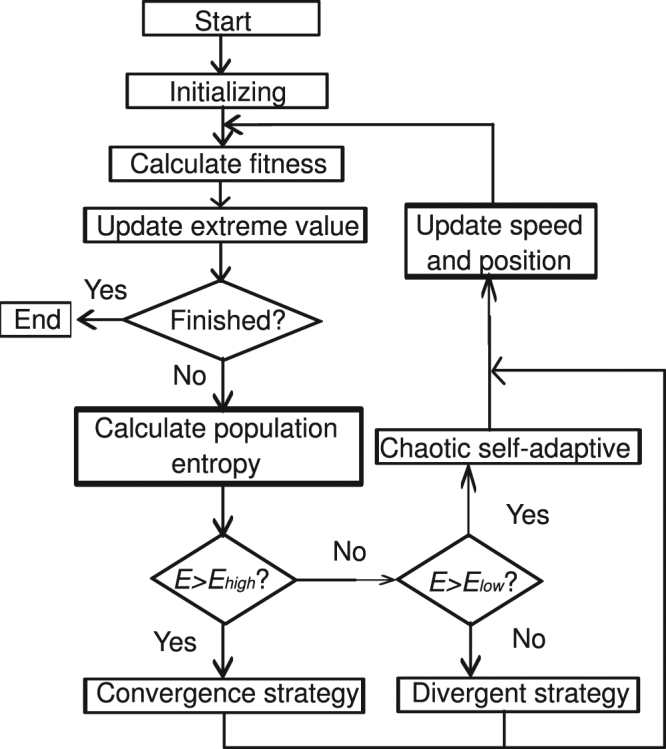
Flowchart of CSAPSO-EDCD algorithm.

Figure 1 shows the CSAPSO-EDCD algorithm flowchart.

### Hybrid Intelligent Model

The relationship between the output and input of the RBF ANN is defined as follow:

11 $$y=f({w}_{h,o},{B}_{h,o},{C}_{basis-fun}),$$

where *W*_*h,o*_ and *B*_*h,o*_$(1\le h\le c)$, $(1\le o\le p)$ are respectively the weight matrix and deviation matrix of the hidden node *h* and the output node *o*; *C*_*basis*−*function*_ is the base function center; is the number of output nodes.

Three parameters of RBF ANN, namely, *c*_*i*_, *σ*_*i*_, *w*_*i*_, are optimized through the above CSAPSO-EDCD algorithm. Thus, the structure of the particle is defined as:

12 $$particle(i)=[{W}_{h,o},{B}_{h,o},{C}_{basis-fun}]$$

RBF ANN was trained by the CSAPSO-EDCD algorithm and the pKa prediction model was developed and called CSAPSO-EDCD RBF ANN.

## Experimental

### Experimental Database

The experimental database of neutral and basic drugs from the previous study^50^ is provided in Table 1. The database, which consists of 74 sets of data, is divided into two subsets by using the random selection method in order to obtain a more reasonable prediction model, and the two subsets denote a training set and a testing set, respectively. The training set is used to establish the model and the testing set is applied to test the performance of the model.

**Table 1:** Experimental database.

No.	Compounds	Experimental pKa
1	ergotamine	6.3
2	nefazodone	6.5
3	nizatidine	6.59
4	trazodone	6.79
5	mirtazapine	7.3
6	clozapine	7.63
7	domperidone	7.9
8	tolamolol	7.9
9	lidocaine	7.94
10	naloxone	7.94
11	quinidine	8.05
12	diltiazem	8.06
13	nicotine	8.1
14	perphenazine	8.11
15	butorphanol	8.19
16	codeine	8.2
17	nebivolol	8.22
18	galanthamine	8.32
19	fentanyl	8.43
20	ranitidine	8.47
21	oxycodone	8.53
22	cocaine	8.7
23	meperidine	8.7
24	timolol	8.8
25	remoxipride	8.9
26	verapamil	8.92
27	rivastigmine	8.99
28	promethazine	9.1
29	mexiletine	9.15
30	levomepromazine	9.19
31	betaxolol	9.21
32	trimipramine	9.24
33	chlorpromazine	9.25
34	chlorpheniramine	9.26
35	propafenone	9.27
36	flecainide	9.3
37	citalopram	9.38
38	clomipramine	9.38
39	labetalol	9.4
40	amitriptyline	9.4
41	propranolol	9.45
42	sumatriptan	9.5
43	venlafaxine	9.5
44	azelastine	9.54
45	pindolol	9.54
46	bisoprolol	9.57
47	alprenolol	9.6
48	acebutolol	9.67
49	nadolol	9.67
50	metoprolol	9.7
51	tacrine	9.8
52	tolterodine	9.8
53	atropine	9.84
54	terbutaline	10
55	atomoxetine	10.1
56	nortriptyline	10.1
57	desipramine	10.23
58	maprotiline	10.5
59	amantadine	10.68
60	cimetidine	6.97
61	sufentanil	7.85
62	clonidine	8.05
63	morphine	8.18
64	risperidone	8.3
65	haloperidol	8.65
66	azithromycin	8.74
67	diphenhydramine	9.1
68	procainamide	9.24
69	promazine	9.28
70	imipramine	9.45
71	paroxetine	9.51
72	atenolol	9.6
73	sotalol	9.76
74	quinacrine	10.2

In this study, the 70% of the data in the database (52 sets of data) were used for training; the remaining data (22 sets of data) were applied to test the model.

### Molecular Descriptors

To establish the relationship between the pKa value and the molecular structure, the molecular structure was indirectly characterized by molecular descriptors. In this paper, molecular descriptors were generated by the following steps. Firstly, the molecular structure was established with Chemdraw UItra 7.0. Secondly, the established molecular structure was optimized with Hyper Chem 7.5. Then, the molecular descriptors were calculated in CODESSA software and 686 molecular descriptors were obtained. In order to reduce the complexity and obtain the most relevant descriptors for the pKa value, the CSAPSO-EDCD algorithm is adopted in the selection of molecular descriptors as follows:

- Step 1: Population initialization. Initialize population size, the max and min number of iterations, etc. and set the population individual as molecular descriptors. Table 2 shows the parameters of the CSAPSO-EDCD algorithm.

**Table 2:** Parameters of the CSAPSO-EDCD algorithm.

Parameters	Descriptions	Values
m	Number of particles	50
it*max*	Iteration times	2000
min*error*	Minimum error	1.00E-07
w	Inertia weight	Self-adaptive
c_1_	Cognitive component	Generated by Lorenz chaotic operator
c_2_	Social component	Generated by Lorenz chaotic operator
- Step 2: Fitness evaluation. Calculate the fitness value of molecular descriptors of each individual.
- Step 3: Update the molecular descriptors. The velocity and position of the particles are updated with the local and global extreme values and the next population of molecular descriptor will be obtained.
- Step 4: Update the fitness of molecular descriptor.
- Step 5: Finished. Confirm whether the iterative conditions are satisfied. If they are satisfied, then the evolution is finished, otherwise jump to Step 2 to continue.

Finally, 5 molecular descriptors are selected by CSAPSO-EDCD algorithm (Table 3).

**Table 3:** Molecular descriptors selected by CSAPSO-EDCD algorithm.

No.	Molecular descriptors	Descriptor types
1	Relative number of N atoms	Constitutional descriptors
2	Randic index (order 3)	Topological descriptors
3	RNCG relative negative charged (QMNEG/QTMINUS) [Quantum-Chemical PC]	Electrostatic descriptors
4	RNCS Relative negative charged SA (SAMNEG * RNCG) [Zefirov’s PC]	Electrostatic descriptors
5	Max net atomic charge	Quantum descriptors

### Model Establishment

The CSAPSO-EDCD RBF ANN model was developed according to the molecular descriptors selected by CSAPSO-EDCD algorithm and consisted of 3 layers: the input layer, hidden layer, and output layer. The input layer has 5 input parameters representing the 5 molecular descriptors, the relative number of N atoms, the Randic index (order 3), the RNCG relative negative charged (QMNEG/QTMINUS) [Quantum-Chemical PC], the RNCS relative negative charged SA (SAMNEG*RNCG) [Zefirov’s PC], and the maximum net atomic charge. The output layer has only 1 output parameter representing the pKa value.

**Figure 2:**
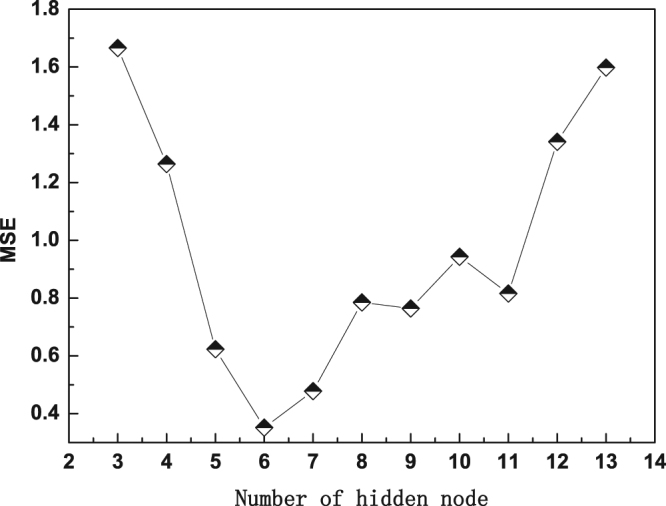
Relationship between MSE and the number of hidden nodes.

Two methods are commonly used to determine the number of nodes in hidden layer: the formula method and the heuristic method. In this study, the two methods were adopted. First, the number of the hidden nodes was calculated through the formula method (2*sqrt (m*n) + 1, where *m* and *n* denote the numbers of input nodes and output layer nodes, respectively). Then, the heuristic method was adopted to confirm the optimal number of hidden nodes. There were 5 input nodes and 1 output node in this study. Therefore, 5 hidden nodes were obtained through the formula method. It is assumed that the number of nodes in hidden layer is explored from 3 to 13. Figure 2 shows the relationship between the prediction error and the number of hidden nodes.

As shown in Fig. 2, with the increase in the number of hidden nodes, MSE decreases firstly and then increases. When the number of hidden nodes is 6, the MSE reaches its minimum value and the structure of the model is optimal. Therefore, the CSAPSO-EDCD RBF ANN model structure is 5-6-1.

### Model Evaluation

The prediction capabilities of different models are evaluated in terms of absolute average relative deviation (*AARD*), root mean square error of prediction (*RMSEP*), and squared correlation coefficient (*R*^2^). The three indicators are defined as follows:

13 $$AARD=\frac{1}{N}\sum _{i=1}^{N}\frac{|{\overline{y}}_{i}-{y}_{i}|}{{y}_{i}};$$

14 $$RMSEP=\sqrt{\frac{1}{N}\sum _{i=1}^{N}({y}_{i}-{\bar{y}}_{i}{)}^{2}};$$

15 $${R}^{2}=\frac{{[\sum _{i=1}^{N}({y}_{i}-{y}_{ave})({\bar{y}}_{i}-{\bar{y}}_{ave})]}^{2}}{\sum _{i=1}^{N}({y}_{i}-{y}_{ave}{)}^{2}\sum _{i=1}^{N}({\bar{y}}_{i}-{\bar{y}}_{ave}{)}^{2}};$$

where *N* is the number of data samples; is the predicted value; *y*_*i*_ denotes the experimental value; *y*_*ave*_ denotes the average of the experimental values; ${\bar{y}}_{ave}$ denotes the average of the predicted values.

## Results and Discussion

### Results of the proposed model

**Figure 3:**
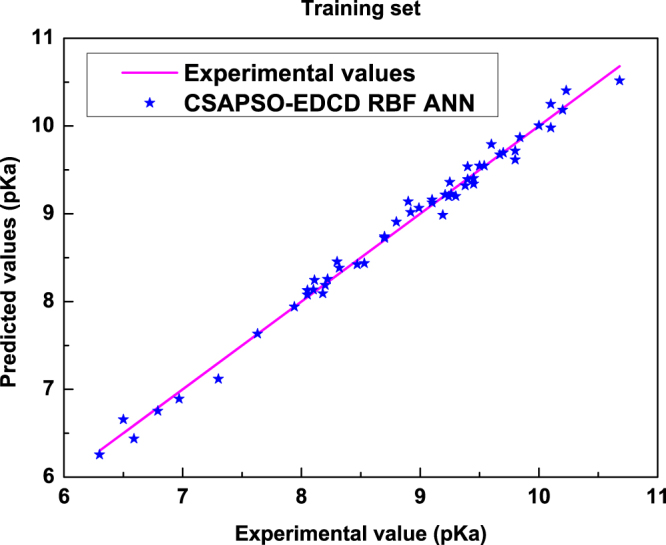
Correlations between predicted values and experimental data in the training set.

Experiments were performed in Windows 7 SP1 64-bit operating system (4.00 GB of memory and 4 Intel (R) Core(TM) i5-4460 CPU @ 3.20 GHz processors). Through Matlab 2010a software programing, a 5-6-1 CSAPSO-EDCD RBF ANN model was proposed to predict the pKa values of 74 neutral and basic drugs. The model was trained with the 52 data points in the training set and tested with the 22 data points in the testing set. Figure 3 shows the correlogram between the experimental data and the predicted values. The straight line denotes the experimental data, whereas the stars denote the predicted values in this paper. The vertical distance between the star-shaped point and the straight line indicates the absolute error between the predicted value and the experimental value.

**Figure 4:**
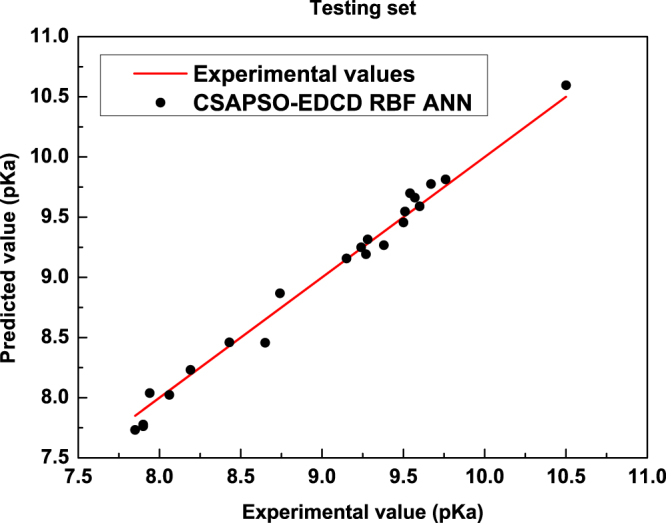
Correlation between predicted values and experimental data in the testing set.

As shown in Fig. 3, in the training set, the prediction values of the model are close to the experimental data. The vertical distances between the star-shaped point and the straight line show that the prediction error of the model is small and that the prediction accuracy is high. Training graph indicates that the training effect of model is better. Figure 4 shows the correlogram between the experimental data and the predicted value in the testing set. The predicted values of the model are consistent with the experimental data in the testing set. Table 4 shows the statistical parameters of the proposed model.

**Table 4:** Statistical parameters of the proposed model.

Sets	*AARD*	*RMSEP*	*R* ^2^
Training	0.2316	0.0263	0.9873
Testing	0.3105	0.0411	0.9685
Average	0.2711	0.0337	0.9779

In the two subsets, the model shows the better comprehensive performance. According to the statistical data, the prediction effect of the proposed model is good and the prediction error is small. Therefore, the comprehensive performance is good. The prediction accuracy and correlation analysis showed that the model had the good prediction performance (Table 4) and the above results confirmed that the prediction performance of the model was excellent.

### Validation analysis with other test databases

Furthermore, in order to verify the robustness and scalability of the proposed model, another testing database containing 20 data points was additionally established for the performance validation. The database from previous studies^51,52^ is provided in Table 5.

**Table 5:** Additional testing database.

NO.	Compounds	Experimental pKa
1	Guanidine	13.8
2	Clomipramine	9.42
3	Papaverine	6.4
4	Clotrimazole	5.75
5	Tryptophan	9.1
6	Methylamine	10.62
7	sec-Butylamine	10.56
8	Imipramine	9.6
9	n-Octylamine	10.7
10	Morpholine	8.5
11	Procaine	9.11
12	Guanethidine	11.4
13	Imidazo[2,3-b]thioxazole	8
14	Trimipramine	9.39
15	Dimethyl-iso-propylamine	10.3
16	tert-Butylcyclohexylamine	11.23
17	Sotalol	9.3
18	Alphaprodine	8.7
19	p-Toluidine	5.1
20	Nikethamide	3.5

**Figure 5:**
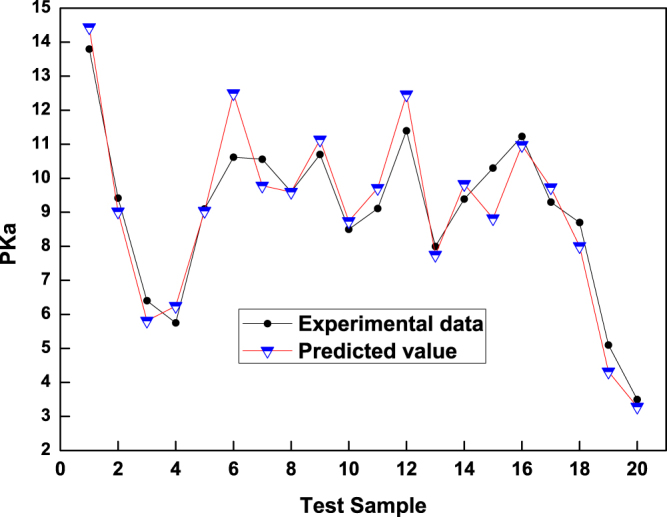
Correlation between predicted values and experimental data in testing database.

Figure 5 shows the correlation between the predicted pKa data and experimental pKa data. The predicted pKa data obtained by the proposed model were well consistent with experimental pKa. Table 6 displays the prediction performance verified with the testing database. The results indicated the superior prediction performance of the proposed model.

**Table 6:** Statistical results of the proposed model in testing database.

	AARD	RMSEP	R^2^
Max	0.9857	0.1005	0.9992
Min	0.1243	0.06337	0.8786
Average	0.6656	0.0742	0.9212

The data in this testing database were not trained by the prediction model. The testing results indicated that the proposed model had the good prediction performance with the better robustness and scalability.

### Comparison of the Proposed Model against Other Models

**Figure 6:**
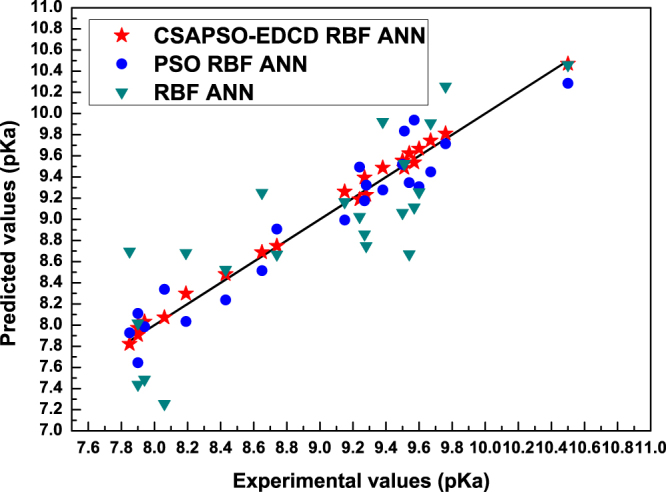
Correlation between prediction and experimental values of the comparison models.

In this paper, to verify the performance of the CSAPSO-EDCD RBF ANN model, we chose two congeneric models (PSO RBF ANN and RBF ANN model) as the comparison models and each model was tested with the testing set. Figure 6 shows the correlation between prediction and experimental values of comparison models.

**Figure 7:**
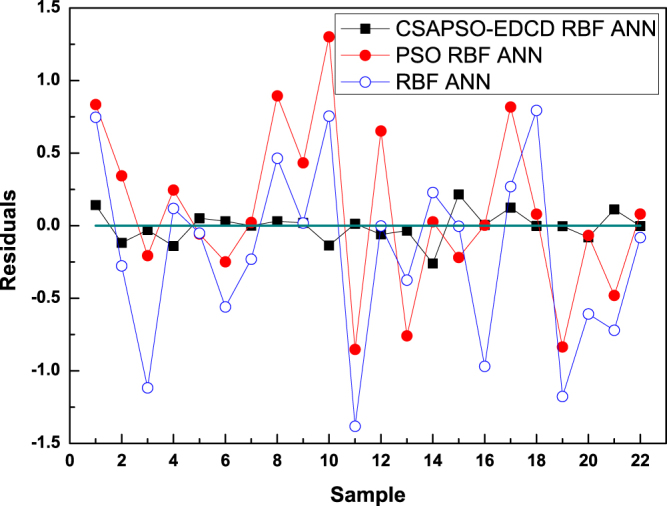
Residual curve for each comparison model.

The vertical distances between the prediction data points and experimental data points showed that the prediction performances of the three models were increased according to the order: RBF ANN, PSO RBF ANN, and CSAPSO-EDCD RBF ANN. PSO RBF ANN is slightly better than RBF ANN. Figure 7 shows the residual curve between the experimental and predicted values of each comparison model. Table 7 shows the statistical parameters of the comparison models.

**Table 7:** Statistical results of each comparison model.

Models	AARD	RMSEP	R^2^	Average calculating time (S)	Average CPU utilization
RBF ANN	1.0892	0.4002	0.8833	36	64%
PSO RBF ANN	0.8898	0.1013	0.8952	57	75%
CSAPSO-EDCD RBF ANN	0.3105	0.0411	0.9685	38	59%

According to the statistical results, the accuracy of the CSAPSO-EDCD RBF ANN model is good. Execution time of the CSAPSO-EDCD RBF ANN model is close to that of the RBF ANN model, and the CPU utilization is less than that of others. In fact, the intervention of intelligent algorithm is bound to consume more computation time. However, the computation time is acceptable because the improved PSO algorithm enhances the training and prediction performances of the model.

### Discussion

#### Discussion of molecule descriptor selection using CSAPSO-EDCD algorithm

Based on the results of molecular descriptor selection, 5 molecular descriptors were adopted based on CSAPSO-EDCD algorithm.

As for the constitutional descriptors, the relative number of N atoms, has a great influence on the density of electron cloud. The relative number of N atoms is generally proportional to the electron cloud density. Therefore, the polarity of the positive and negative charges in the molecule becomes larger and the pKa value is smaller. The relative number of N atoms can be used to characterize the constituent of the molecular structure.

As for the topological descriptor, the Randic index (order 3) indicates the size and shape of a molecule, or the degree of branching, and shows the molecular dispersion force. The molecular volume increases with the increase in the dispersion force of the molecule, thus leading to the decrease in the pKa value. The Randic index (order 3) can better characterize the topological structure of the molecule.

As for the electrostatic descriptor, the RNCG relative negative charged (QMNEG/QTMINUS) [Quantum-Chemical PC] and RNCS Relative negative charged SA (SAMNEG * RNCG) [Zefirov’s PC] depend on the distribution of the calculated electric charge on the molecule. The negative coefficient of relative negative charge shows an inverse relationship with pKa value. In terms of the relative negative charge surface area, the probability of the positive ion to replace the proton shows an inverse relationship with the most negative atom solvent contact area and pKa value. The relative negative charge and its surface area can be used to characterize the molecular electrostatic parameters.

As for the quantum chemical descriptors, the maximum net atomic charge is related to the polarization action and directly proportional to the pKa value. Simultaneously, it can also be used to characterize the quantum chemical structure of molecules.

According to the results of molecular descriptors, the molecular descriptors selected by the CSAPSO-EDCD algorithm can better characterize the molecular structure and reflect the close relationship between the structures of various drug compounds and the pKa value.

#### Discussion of the model of CSAPSO-EDCD RBF ANN

The CSAPSO-EDCD algorithm can effectively overcome the shortcomings of the PSO. It can provide an efficient training algorithm for RBF ANN through the combination of chaotic self-adaptive, the diversity of population entropy and the convergence/divergence strategies. The good performance is ascribed to the CSAPSO-EDCD algorithm for selecting molecular descriptors and training RBF ANN.

The QSAR model based on the hybrid intelligent method has the less computation load and good prediction performance. If the molecular structure is unknown, the structure activity relationship can be predicted accurately and effectively. However, the model is established based on data training and obviously affected by the training data. Moreover, the physical meaning of the structure-activity relationship is one of the most important challenges.

## Conclusions

The CSAPSO-EDCD RBF ANN model can accurately predict the pKa values of various drug molecules. The model shows the good performance in predicting the pKa values of neutral and basic drugs and its prediction accuracy and correlation are higher. The model can provide a reference for QSAR modeling.

The molecular descriptors selected by the CSAPSO-EDCD algorithm can better characterize the molecular structure and provide ideas for the selection of molecular descriptors in QSAR.

## References

[1] Zhang C (2016). In silico Prediction of Drug Induced Liver Toxicity Using Substructure Pattern Recognition Method. Mol. Inf..

[2] Gebreyohannes S, Dadmohammadi Y, Neely BJ, Gasem KAM (2016). A Comparative Study of QSPR Generalized Activity Coefficient Model Parameters for Vapor-Liquid Equilibrium Mixtures. Ind. Eng. Chem. Res..

[3] Romand S, Schappler J, Veuthey JL, Carrupt PA, Martel S (2014). cIEF for rapid pK(a) determination of small molecules: A proof of concept. Eur. J. Pharm. Sci..

[4] Settimo L, Bellman K, Knegtel RMA (2014). Comparison of the Accuracy of Experimental and Predicted pKa Values of Basic and Acidic Compounds. Pharmaceut. Res..

[5] Sliwoski G, Mendenhall J, Meiler J (2016). Autocorrelation descriptor improvements for QSAR: 2DA_Sign and 3DA_Sign. J. Comput. Aid. Mol. Des..

[6] Yu HY (2015). Modeling and predicting pK(a) values of mono-hydroxylated polychlorinated biphenyls (HO-PCBs) and polybrominated diphenyl ethers (HO-PBDEs) by local molecular descriptors. Chemosphere..

[7] Rojas C (2016). Quantitative structure-activity relationships to predict sweet and non-sweet tastes. Theor. Chem. Acc..

[8] Fujita T, Winkler DA (2016). Understanding the Roles of the “Two QSARs”. J. Chem. Inf. Model..

[9] Wang X, Luo F, Qian Y, Ranzi G (2016). A Personalized Electronic Movie Recommendation System Based on Support Vector Machine and Improved Particle Swarm Optimization. Plos One..

[10] Pedretti, G. *et al*. Memristive neural network for on-line learning and tracking with brain-inspired spike timing dependent plasticity. *Sci. Rep*. **7** (2017). 10.1038/s41598-017-05480-0 PMC5509735 28706303

[11] Barron LP, McEneff GL (2016). Gradient liquid chromatographic retention time prediction for suspect screening applications: A critical assessment of a generalised artificial neural network-based approach across 10 multi-residue reversed-phase analytical methods. Talanta..

[12] Zhou W (2016). High-accuracy QSAR models of narcosis toxicities of phenols based on various data partition, descriptor selection and modelling methods. RSC. Adv..

[13] Guo, J. *et al*. Application of artificial neural network to investigate the effects of 5-fluorouracil on ribonucleotides and deoxyribonucleotides in HepG2cells. *Sci. Rep*. **5** (2015). 10.1038/srep16861 PMC4649619 26578061

[14] Liu, S. *et al*. Differentiating Thamnocalamus Munro from Fargesia Franchet emend. Yi (Bambusoideae, Poaceae): novel evidence from morphological and neural-network analyses. *Sci. Rep*. **7** (2017). 10.1038/s41598-017-04613-9 PMC5482892 28646152

[15] Wang NN (2016). ADME Properties Evaluation in Drug Discovery: Prediction of Caco-2 Cell Permeability Using a Combination of NSGA-II and Boosting. J. Chem. Inf. Model..

[16] Polanski J, Walczak B (2000). The comparative molecular surface analysis (COMSA): a novel tool for molecular design. Comput. Chem..

[17] Luan F (2005). Prediction of retention time of a variety of volatile organic compounds based on the heuristic method and support vector machine. Anal. Chim. Acta..

[18] Bianchi, F. M., Livi, L., Alippi, C. & Jenssen, R. Multiplex visibility graphs to investigate recurrent neural network dynamics. *Sci. Rep*. **7** (2017). 10.1038/srep44037 PMC5345088 28281563

[19] Liu, Z., Gao, J., Yang, G., Zhang, H. & He, Y. Localization and Classification of Paddy Field Pests using a Saliency Map and Deep Convolutional NeuralNetwork. *Sci. Rep*. **6** (2016). 10.1038/srep20410 PMC4750055 26864172

[20] Li M (2015). Solubility prediction of supercritical carbon dioxide in 10 polymers using radial basis function artificial neural network based on chaotic self-adaptive particle swarm optimization and K-harmonic means. RSC. Adv..

[21] Li MS (2013). Prediction of gas solubility in polymers by back propagation artificial neural network based on self-adaptive particle swarm optimization algorithm and chaos theory. Fluid. Phase. Equilibr..

[22] Azad FN (2016). Optimization of the process parameters for the adsorption of ternary dyes by Ni doped FeO(OH)-NWs-AC using response surface methodology and an artificial neural network. RSC. Adv..

[23] Li M, Wu W, Chen B, Wu Y, Huang X (2017). Solubility prediction of gases in polymers based on an artificial neural network: a review. RSC. Adv..

[24] Cano G (2017). Automatic selection of molecular descriptors using random forest: Application to drug discovery. Expert. Syst. Appl..

[25] Zhou YW, Wu JM, Xu X (2016). Improving B3LYP Heats of Formation with Three-Dimensional Molecular Descriptors. J. Comput. Chem..

[26] Sahoo S, Adhikari C, Kuanar M, Mishra BK (2016). A Short Review of the Generation of Molecular Descriptors and Their Applications in Quantitative Structure Property/Activity Relationships. Curr. Comput.-Aided Drug Des..

[27] Gao, Y., Du, W. & Yan, G. Selectively-informed particle swarm optimization. *Sci. Rep*. **5** (2015). 10.1038/srep09295 PMC4365407 25787315

[28] Lombardo F, Jing YK (2016). In Silico Prediction of Volume of Distribution in Humans. Extensive Data Set and the Exploration of Linear and Nonlinear Methods Coupled with Molecular Interaction Fields Descriptors. J. Chem. Inf. Model..

[29] Yousefinejad S, Hemmateenejad B (2015). Chemometrics tools in QSAR/QSPR studies: A historical perspective. Chemometr. Intell. Lab Syst..

[30] Zafar A, Reynisson J (2016). Hydration Free Energy as a Molecular Descriptor in Drug Design: A Feasibility Study. Mol. Inf..

[31] Shen L (2016). A novel local manifold-ranking based K-NN for modeling the regression between bioactivity and molecular descriptors. Chemometr. Intell. Lab Syst..

[32] Shahlaei M (2013). Descriptor Selection Methods in Quantitative Structure-Activity Relationship Studies: A Review Study. Chem. Rev..

[33] Charifson PS, Walters WP (2014). Acidic and Basic Drugs in Medicinal Chemistry: A Perspective. J. Med. Chem..

[34] Chakravorty A, Jia Z, Li L, Alexove E (2017). A New DelPhi Feature for Modeling Electrostatic Potential around Proteins: Role of Bound Ions and Implications for Zeta-Potential. Langmuir..

[35] Petukh M, Stefl S, Alexov E (2013). The Role of Protonation States in Ligand-Receptor Recognition and Binding. Curr. Pharm. Design..

[36] Onufriev AV, Alexov E (2013). Protonation and pK changes in protein-ligand binding. Q. Rev. Biophys..

[37] Peng YH, Alexov E (2017). Computational investigation of proton transfer, pKa shifts and pH-optimum of protein-DNA and protein-RNA complexes. Proteins-Structure Function and Bioinformatics..

[38] Li L, Chakravorty A, Alexov E (2017). DelPhiForce, a Tool for Electrostatic Force Calculations: Applications to Macromolecular Binding. J. Comput. Chem..

[39] Li, L., Alper, J. & Alexov, E. Multiscale method for modeling binding phenomena involving large objects: application to kinesin motor domains motion along microtubules. *Sci. Rep*. **6** (2016). 10.1038/srep23249 PMC4796874 26988596

[40] Riffault-Valois L (2017). Molecular Fingerprint Comparison of Closely Related Rose Varieties based on UHPLC-HRMS Analysis and Chemometrics. Phytochem. Anal..

[41] Muegge I, Mukherjee P (2016). An overview of molecular fingerprint similarity search in virtual screening. Expert. Opin. Drug. Discovery..

[42] Cereto-Massague A (2015). Molecular fingerprint similarity search in virtual screening. Methods..

[43] Petersen CR (2014). Mid-infrared supercontinuum covering the 1.4–13.3 mu m molecular fingerprint region using ultra-high NA chalcogenide step-index fibre. Nat. Photonics..

[44] Zhang YX (2014). An improved QSPR method based on support vector machine applying rational sample data selection and genetic algorithm-controlled training parameters optimization. Chemometr. Intell. Lab Syst..

[45] Tsekouras GE, Tsimikas J (2013). On training RBF neural networks using input-output fuzzy clustering and particle swarm optimization. Fuzzy Set. Syst..

[46] Kennedy, J. & Eberhart, R., presented at the Proceedings of the 1995 IEEE International Conference on Neural Networks. Part 1 (of 6), Perth, Aust, (unpublished) (1995).

[47] Eberhart, R. & Kennedy, J., presented at the Proceedings of the 1995 6th International Symposium on Micro Machine and Human Science, October 4, 1995 - October 6, 1995, Nagoya, Jpn, (unpublished) (1995).

[48] Zhao XL, Turk M, Li W, Lien KC, Wang GZ (2016). A multilevel image thresholding segmentation algorithm based on two-dimensional K-L divergence and modified particle swarm optimization. Appl. Soft. Comput..

[49] Taghiyeh S, Xu J (2016). A new particle swarm optimization algorithm for noisy optimization problems. SWARM. INTELL..

[50] Luan F (2005). Prediction of pK(a) for neutral and basic drugs based on radial basis function neural networks and the heuristic method. Pharmaceut. Res..

[51] Jensen JH, Swain CJ, Olsen L (2017). Prediction of pK(a) Values for Druglike Molecules Using Semiempirical Quantum Chemical Methods. J. Phys. Chem. A..

[52] Eckert F, Klamt A (2006). Accurate prediction of basicity in aqueous solution with COSMO-RS. J. Comput. Chem..

